# Efficient Color Quantization Using Superpixels

**DOI:** 10.3390/s22166043

**Published:** 2022-08-12

**Authors:** Mariusz Frackiewicz, Henryk Palus

**Affiliations:** Department of Data Science and Engineering, Silesian University of Technology, Akademicka 16, 44-100 Gliwice, Poland

**Keywords:** color quantization, superpixel, image quality, clustering, computation rate

## Abstract

We propose three methods for the color quantization of superpixel images. Prior to the application of each method, the target image is first segmented into a finite number of superpixels by grouping the pixels that are similar in color. The color of a superpixel is given by the arithmetic mean of the colors of all constituent pixels. Following this, the superpixels are quantized using common splitting or clustering methods, such as median cut, k-means, and fuzzy c-means. In this manner, a color palette is generated while the original pixel image undergoes color mapping. The effectiveness of each proposed superpixel method is validated via experimentation using different color images. We compare the proposed methods with state-of-the-art color quantization methods. The results show significantly decreased computation time along with high quality of the quantized images. However, a multi-index evaluation process shows that the image quality is slightly worse than that obtained via pixel methods.

## 1. Introduction

Each pixel within a typical color digital image can display one of up to 16,777,216 different colors. For convenience, many approaches to the processing of color digital images, such as image compression or image segmentation, operate across a much smaller number of image colors. The use of such a reduced set of image colors is known as color quantization (CQ). Such approaches attempt to maintain maximum similarity between the quantized image and the original image, while reducing the number of colors.

Broadly, three groups of color quantization methods can be distinguished [1,2]. The first group includes splitting techniques, such as median cut (MC) [3], octree [4], and Wu’s algorithm [5]. These techniques iteratively split the RGB color solid into smaller boxes, the mean values of which form the colors of the resulting palette. Such approaches have the benefit of speed; however, the resulting images often contain colors that are substantially different from those of the original image.

The second group of methods uses pixel clustering algorithms, such as k-means (KM) [6] and fuzzy c-means (FCM) [7,8]. Such data clustering techniques have a wide range of applications outside of color digital image processing. During the clustering process, pixels within the target image are assigned to clusters in 3-D color space. The clusters are constructed based on a given similarity criterion, such as pixel color or location within the image. The clustering of a color image requires the unsupervised classification of hundreds of thousands of pixels or more, based on their color similarity.

As such, these techniques provide high quality results but suffer from long computation times due to high computational complexity. This time can be reduced with the application of appropriate data structures, and hence a reduction in the number of computed distances between data. Bin Zhang et al. [9] proposed the k-harmonic means clustering technique. Extending KM, this technique minimizes the objective function by calculating the harmonic mean of the distances of points from the cluster centers.

Frackiewicz and Palus [10] demonstrated the application of k-harmonic means to color quantization. The third group of methods consists of additional CQ techniques that primarily exist within the field of artificial intelligence. These techniques include neural networks, such as the Kohonen network NeuQuant [11] and Neural Gas [12], and metaheuristic methods based on the flocking behavior of animals, such as ants [13,14], bees [2], fireflies [15], and frogs [16]. Typically, the use of these techniques for CQ is somewhat time consuming.

Note that the image quality resulting from CQ processes can be improved by considering spatial features, such as dithering based on diffusion errors [17]. The above CQ methods either produce low quality images or consume excessive processing time. The goal of further work in this area is to obtain high quality quantized images at a low computational cost.

Ren and Malik [18] first proposed the use of superpixel image representations in 2003. A single superpixel replaces a group of pixels with similar characteristics, such as intensity, color, and texture. Superpixels provide a convenient and compact image representation for computationally complex tasks. A given image has fewer superpixels than pixels; hence, algorithms can run faster by operating on superpixels instead of pixels. Moreover, pixels themselves carry no visual information; superpixels are matched to the image content and hence contain such information. Each superpixel region is assigned an average color value that represents its constituent pixels.

Additionally, superpixels preserve the majority of image boundaries. Superpixel generators construct a given number of superpixels with specified properties, such as compactness or size. From a CQ viewpoint, the color of the pixel plays the most important role. The same is true when quantizing color on superpixels, while the shape features of superpixels play a secondary role in CQ. Superpixel generation is a useful tool for image preprocessing as it segments an image into small uniform regions. Such partitioning can improve the efficiency of subsequent processes, such as CQ and image segmentation.

Approaches to superpixel image segmentation include clustering, gradient, graph, and watershed algorithms [19,20]. Among such approaches, the Simple Linear Iterative Clustering (SLIC) method is the most popular [21]. The SLIC algorithm is based on fast KM, and, classifies pixels by color and location within the image to determine the number of resultant superpixels. Color distance is calculated in perceptually uniform CIELab color space. New modifications to the SLIC algorithm are constantly appearing that improve the quality of superpixel segmentation, for example SLIC++ [22] used for semi-dark images.

Superpixels provide a useful framework for image processing operations such as low-light image enhancement [23], image segmentation [24], saliency detection [25], dimensional reduction in hyperspectral image classification [26], and full-reference image quality assessment [27]. Recently, superpixel algorithms have also been applied to video sequences, for example, to avoid a dimensional explosion problem [28]. Most superpixel applications described in the literature use the SLIC algorithm.

To the best of our knowledge, no existing work uses superpixels for color image quantization. Our novel contribution is the successful use of superpixels for CQ. Such methods can provide a lower computation time than pixel-based methods.

The rest of this paper is organized as follows. Section 2 describes our proposed superpixel CQ methods, analyzes their advantages and presents the image quality indices used in this paper. Section 3 shows the experimental results. Finally, Section 4 concludes the paper.

## 2. Materials and Methods

**Figure 1:**
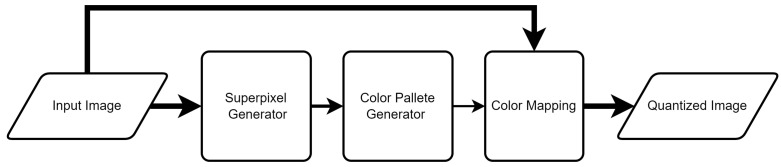
General concept of the proposed superpixel CQ methods.

The proposed superpixel CQ methods, outlined in Figure 1, use the original image as an input to the superpixel generator (in our case SLIC) and an input to the color pixel mapping. Generation of the color palette—the most complex stage of the CQ method—uses only the superpixel image. This approach should significantly reduce the computation time and should shorten the CQ process.

We use three different algorithms to generate color palettes. The MC method creates a cuboid within the RGB color space containing all the points that correspond to the image colors. The algorithm recursively divides the cuboid with orthogonal planes into *k* smaller cuboids. During each step, the largest cuboid is subdivided perpendicular to its longest side at the median point, creating two cuboids containing approximately the same number of pixels. For each of the *k* cuboids, the algorithm then calculates the arithmetic mean of each RGB component across all colors for each cuboid. Within the color palette, the resultant color thus represents the pixels contained in the corresponding cuboid. To complete the process, the color of each pixel within the original image is mapped to its closest color from the palette.

CQ can be treated as a pixel clustering problem—the objective is to form clusters that best represent the image colors. The number *k* of such clusters is given by the target number of colors within the image following quantization. Typically, *k* is even: $k=2n,n\in Z^{+}$. The KM clustering algorithm distributes points among *k* independent clusters, where *k* is predefined. In the original KM implementation, the positions of the *k* initial clustering centers are chosen randomly among all points.

The cluster to which a given point belongs is determined by the distance of that point from each of the cluster centers: a point is assigned to the cluster to which it is closest. These cluster positions are then shifted iteratively, with each cluster center being defined by the arithmetic mean of the points assigned to the cluster. The algorithm stops once a preset number of iterations are completed or once the distance by which each cluster center is moved is below a predefined threshold.

A major drawback of KM is the dependence of the obtained result on the initialization of the clustering process. The quality of the result is highly influenced by the initial positions of the cluster centers. For most applications using KM, the cluster centers are initialized randomly. For example, when applied to an image, pixels are selected randomly within an image. However, this can lead to poor results, and some initialization methods can result in empty clusters.

Deterministic fast splitting initialization techniques, such as MC or Wu’s algorithm [29] show improved results. Further improvement of KM initialization is desirable; initialization should provide high quality clustering within a small number of iterations. Empty clusters should not be generated, as they reduce the number of colors in the quantized image. Vassilvitskii and Arthur [30] proposed the KM++ technique, which performs more strongly than Forgy’s random method for color quantization [31]. The original KM method has been applied to CQ problems throughout the 21st century [32,33,34].

The FCM algorithm is an adaptation of KM that allows each point to belong to multiple clusters. The degree to which a given point has fuzzy membership of a given cluster lies in the range [0, 1]. The higher the fuzzy membership, the stronger the association of a point with a cluster. Therefore, points on the periphery of a cluster are assigned lower degrees of membership than points located at the center of the cluster. Moreover, FCM defines a hyperparameter *m* that determines the level of fuzziness of all clusters. A larger *m* gives fuzzier clusters.

Typically, *m* is set to 2. The center of a given cluster is calculated as the arithmetic mean of all points, weighted by their degree of membership to that cluster. This method is slower than KM, and the results obtained by it still depend on the cluster initialization. Like the KM algorithm, FCM minimizes the intra-cluster variance and maximizes the inter-cluster variance. Both techniques converge to local minima. The FCM method has seen previous applications to CQ problems [35,36,37].

Clustering algorithms, such as KM and FCM, require that the distance from each pixel to every cluster center is calculated. This leads to high computational complexity, particularly for high-resolution images. Introducing superpixels into the CQ algorithm would reduce the computational complexity while producing only a small decrease in image quality.

We present modified, superpixel versions of these algorithms: SPMC, SPKM, and SPFCM. We compare these algorithms with their original counterparts. In particular, we consider the relationship between the number of generated superpixels $N_{SP}$ and the quality of the final results. $N_{SP}$ cannot be less than the final number of colors *k* in the palette. To determine $N_{SP}$, we propose the following empirical formula:

(1) $$N_{SP}=k+SP\_Ratio\cdot k,$$

where SP_Ratio∈{2,4,8,16}.

**Figure 2:**
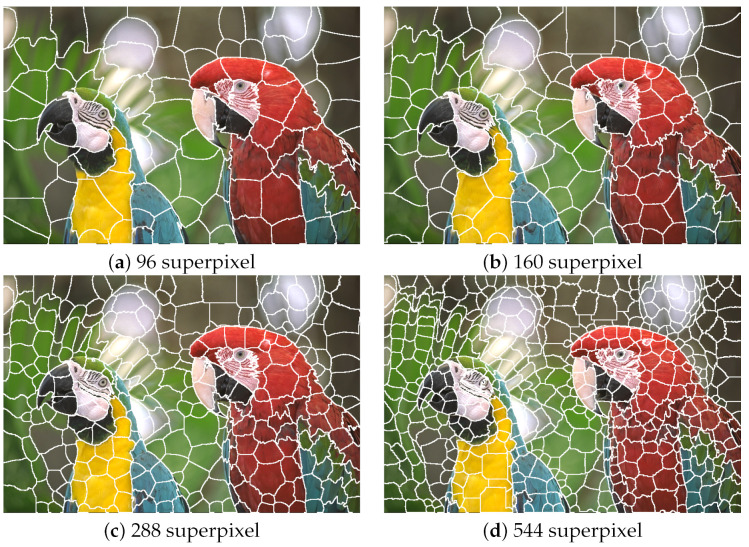
Superpixel generation for image kodim23 with *k* = 32.

The application of this formula to the superpixel generation process is illustrated in Figure 2. Using a quantization level of *k* = 32, four images divided into 96, 160, 288, and 544 superpixels were generated from the source image.

Methods for the evaluation of image quality primarily concern images with specific distortions. Comparatively little research has been undertaken into the evaluation of image quality following distortions caused by CQ [38]. To verify the image quality, we considered the use of nine different image quality indices, including the peak signal-to-noise ratio (PSNR), structural similarity index measure (SSIM), and weighted signal-to-noise ratio (WSNR). Each of these indices could be considered equally suitable for application to CQ image distortions. Recent research has proposed indices more strongly correlating with the human visual system.

Ultimately, for this work, we chose to use the traditional PSNR index alongside the following perceptual quality indices: the color feature similarity index (FSIMc) [39], the directional statistics color similarity index (DSCSI) [40], and the Haar wavelet perceptual similarity index (HPSI) [41]. The usefulness of the latter indices within the CQ domain is already proven [42]. We also use the superpixel similarity (SPSIM) index—one of two indices proposed for application to images segmented by superpixels [27,43].

With the above tools, we can generate superpixel images, perform CQ, and objectively evaluate image quality following quantization, thereby, allowing us to assess and evaluate superpixel CQ methods. All experiments were conducted on a desktop computer with a 3.4 GHz Intel Core i5-8250U CPU, and 20 GB of RAM. The scripts were implemented in the Matlab R2019b environment.

## 3. Experiments and Results

### 3.1. Testing of the Proposed Methods

**Figure 3:**
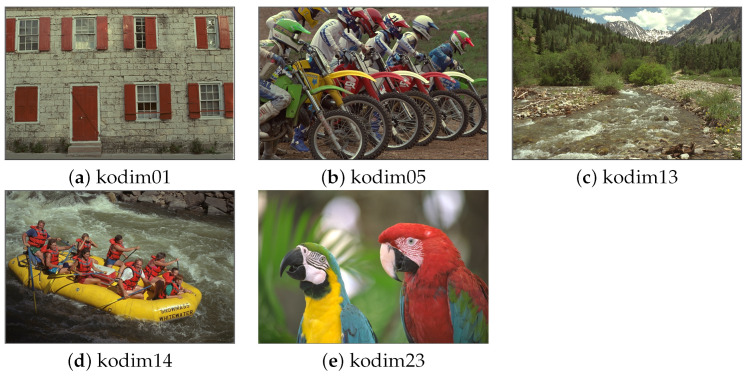
Selected Kodak test images.

For the first set of experiments, we used five images selected randomly from the Kodak image dataset [44]: kodim01, kodim05, kodim13, kodim14, and kodim23. Shown in Figure 3, each image has a spatial resolution of 768 × 512 pixels. We applied CQ to each of these images using all of the described pixel and superpixel methods, with *k* values of 8, 16, 32, 64, 128, and 256. We set *SP_Ratio* = 16. We used the following five indices to evaluate image quality following quantization: PSNR, FSIMc, DSCSI, HPSI, and SPSIM. The value of each index correlates positively with the image quality. We performed a pairwise comparison of each of the three superpixel QC methods with its corresponding pixel method.

**Figure 4:**
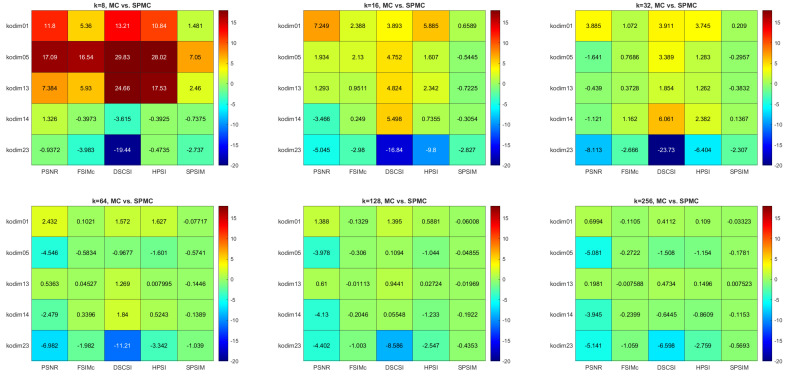
Multi−index quality assessment of quantized images: MC versus SPMC.

**Figure 5:**
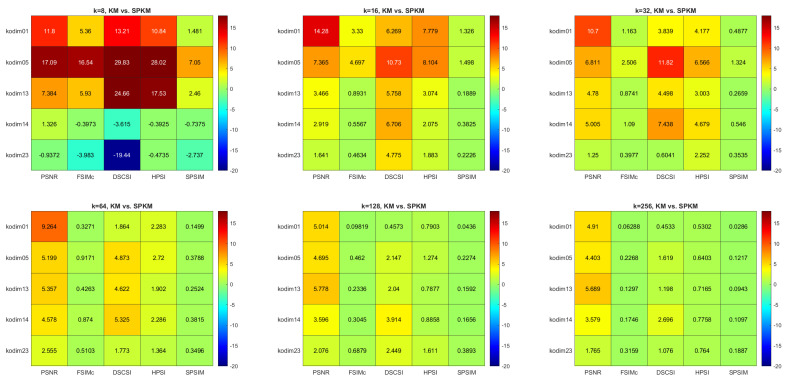
Multi−index quality assessment of quantized images: KM versus SPKM.

**Figure 6:**
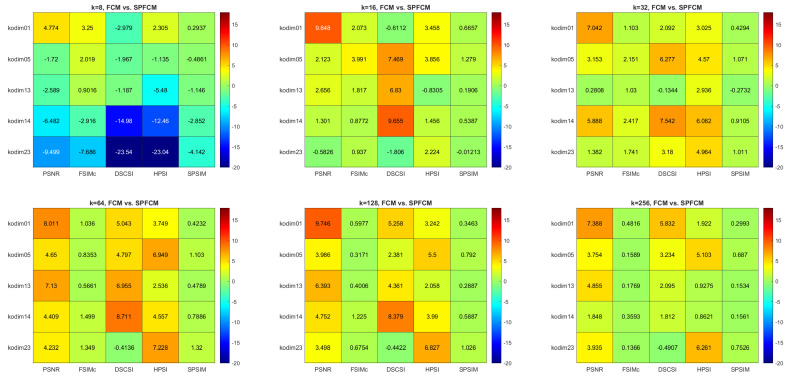
Multi−index quality assessment of quantized images: FCM versus SPFCM.

Figure 4, Figure 5 and Figure 6 present the comparison results for MC versus SPMC, KM versus SPKM, and FCM versus SPFCM, respectively. Each result is presented both numerically, and in the form of a color map, for which the color scale corresponds with the percentage different in quality index between the two methods under comparison. The results are predominantly green, particularly for MC versus SPMC, which indicates no difference in image quality following quantization.

Red indicates that the modified superpixel methods produce images of lower quality than the corresponding pixel methods. Blue indicates that the superpixel methods produce higher quality images than the pixel methods. Smaller values of *k* produce more variable results, with the color maps displaying more red and blue. A comparison of the different quality indices reveals that SPSIM provides the most stable assessment, which is to be expected when applied to superpixel CQ methods.

**Figure 7:**
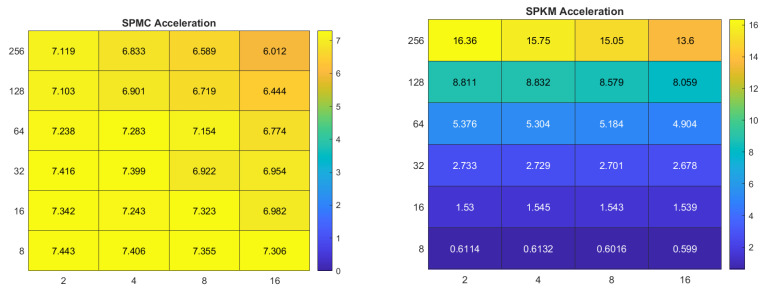

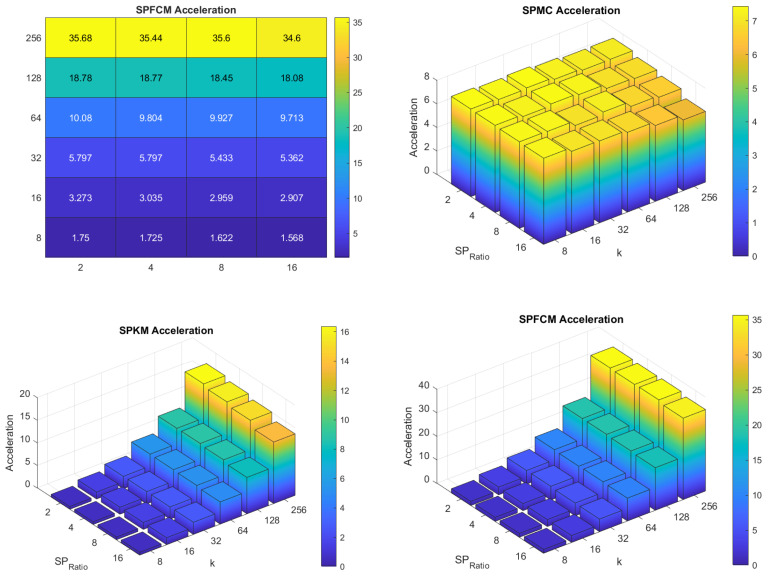
Computation rates of superpixel CQ algorithms relative to the corresponding pixel algorithms.

Figure 7 compares the computation rates of the three superpixel CQ methods with those of the pixel methods. The values shown are averages measured across all five tested images. The SPMC method performs several times faster than the MC method. For the SPKM and SPFCM methods the effect is even more pronounced, with the superpixel methods running up to 15- and 30-times faster, respectively. An exception is the SPKM algorithm with k=8, which performs more slowly than KM. The *SP_Ratio* parameter, which is used to set the number of superpixels, has a negligible impact on the computation rate. Conversely, the value of *k* has a substantial impact. This impact is most strongly seen with the SPKM and SPFCM methods.

### 3.2. The Impact of Image Resolution on Computation Rate

**Figure 8:**
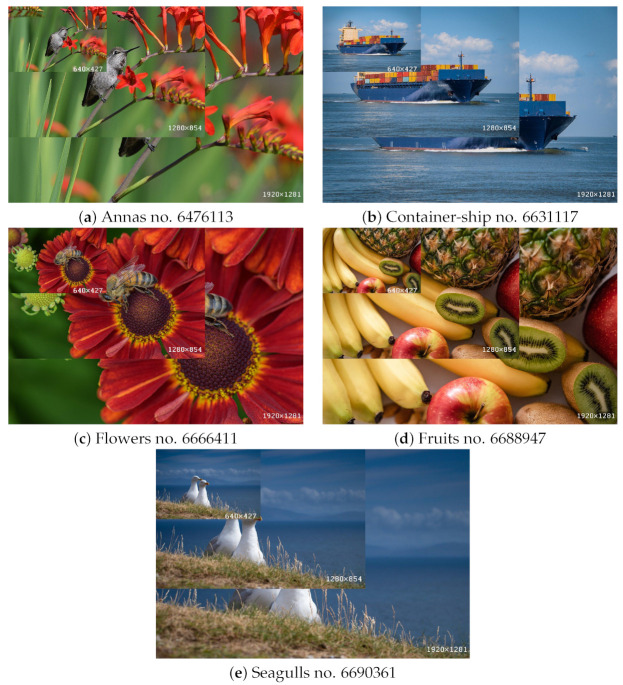
Selected Pixabay test images in three different resolutions: 640 × 427, 1280 × 854, and 1920 × 1281.

To investigate the relationship between image resolution and computation rate, we used a further five images from the Pixabay dataset [45], shown in Figure 8: Annas no. 6476113, Container-ship no. 6631117, Flowers no. 6666411, Fruits no. 6688947, and Seagulls no. 6690361. Each of the five images was available in three different resolutions: 640 × 427, 1280 × 854, and 1920 × 1281.

**Figure 9:**
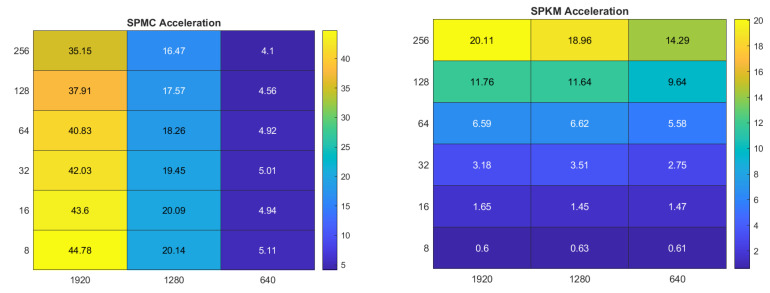

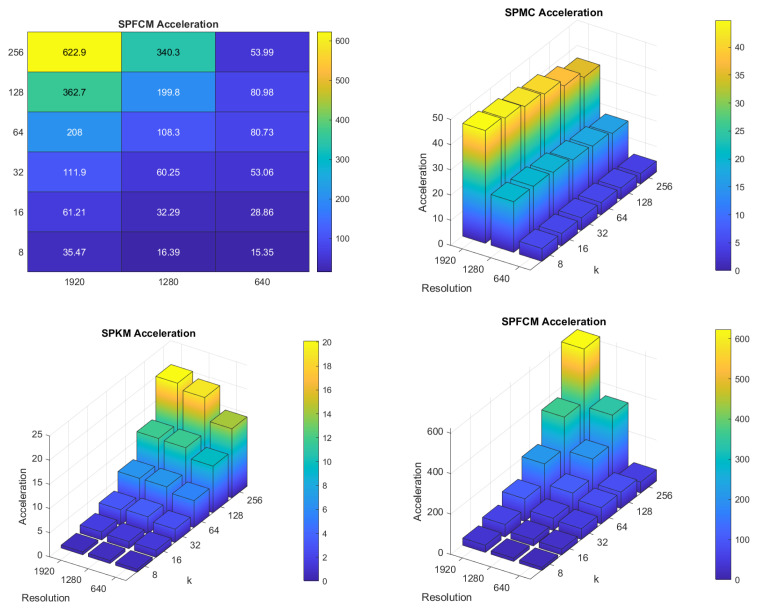
Computation rates of superpixel CQ algorithms for images of different resolution.

Figure 9 presents a comparison of computation rates for each image resolution. The results obtained for the lower resolution images are similar to those produced when using the Kodak images: the SPFCM method provides the highest computation rate; the SPMC method provides the lowest. However, for the higher resolution images an even larger increase in computation rate is observed: up to 340-fold for the medium-resolution images and 623-fold for the high-resolution images. These results validate the application of superpixel CQ methods to megapixel images.

## 4. Conclusions

In this paper, we proposed three superpixel algorithms for color image quantization. These algorithms use superpixels in lieu of the pixels used by classical CQ algorithms. This approach resulted in a many-fold increase in the computation rate, except in the case of the SPKM algorithm with k=8, with minimal degradation of the image quality following quantization.

These outcomes were particularly notable when using the clustering algorithms SPKM and SPFCM, and when applied to high-resolution images. Such algorithms will allow CQ to be performed even in the case of limited computer memory resources. We validated the image quality using multiple image quality indices. A key parameter of the presented superpixel algorithms is the number of superpixels used, which is dependent only on the number of color quantization levels. The optimal choice of superpixel number will be the subject of further research.

## Data Availability

Not applicable.

## Abbreviations

The following abbreviations are used in this manuscript:

CQ	Color quantization
CIELab	Lab color space defined by International Commission on Illumination
DSCSI	Directional statistics color similarity index
FCM	fuzzy c-means
FSIMc	Color feature similarity index
HPSI	Haar wavelet perceptual similarity index
KM	k-means
KM++	k-means++
MC	Median cut
PSNR	Peak signal-to-noise ratio
RGB	Red, green, blue
SLIC	Simple linear iterative clustering
SLIC++	Simple linear iterative clustering++
SPFCM	Superpixel version of fuzzy c-means
SPKM	Superpixel version of k-means
SPMC	Superpixel version of Median cut
SPSIM	Superpixel similarity index
SSIM	Structural similarity index measure
WSNR	Weighted signal-to-noise ratio
